# The authors reply: Letter on: "Prevalence of depression in patients with sarcopenia and correlation between the two diseases: systematic review and meta‐analysis" by Li et al.

**DOI:** 10.1002/jcsm.13008

**Published:** 2022-05-01

**Authors:** Zhenzhen Li, Jirong Yue

**Affiliations:** ^1^ Health Management Center, National Clinical Research Center for Geriatrics, West China Hospital/West China School of Medicine Sichuan University Chengdu China; ^2^ Department of Geriatrics and National Clinical Research Center for Geriatrics, West China Hospital/West China School of Medicine Sichuan University Chengdu Sichuan China

We thank Zhang *et al*.^1^ for their interest and sharing views on our recently published article. Their letter raises several interesting issues that we would like to respond to.

First, we rechecked the data and confirmed that our data was correct.^1^ In Olgun Yazar's study, the sample size of sarcopenia and depression is shown in Table 2 (Supporting Information, *Figure* S1), which shows Control 2 and GD (geriatric depression) group data in terms of sarcopenia stages, with 165 individuals in the control 2 group and 116 patients in the GD group. In the control 2 group, there were four patients with pre‐sarcopenia, nine patients with sarcopenia, nine patients with severe sarcopenia. There were six patients with pre‐sarcopenia, 11 patients with sarcopenia, 21 patients with severe sarcopenia in the GD group. ‘In our analysis, study population involving individuals with sarcopenia, defined as the presence of low muscle mass (LMM), low muscle strength (LMS), and/or low physical performance (LPP).’^2^ Therefore, we excluded pre‐sarcopenia, and the sample size of sarcopenia was: 9 + 9 + 11 + 21 = 50.^2^ In Hsu's study, according to the text description in the results part (*Figure* S2), there were 109 people with sarcopenia. The prevalence of depression was 29.8%, so the number of people with depression was 32 (109*29.8%). At the same time, Table 2 indicates that the study subjects are missing, 28 patients with depression (29.8% of patients with sarcopenia) and 66 patients without depression (70.2% of patients with sarcopenia) (*Figure* S3). Thus, there are 94 sarcopenia patients, and the different sets of data from the original study are shown in *Table* 1. Since we calculated the prevalence, whichever collection of data was used did not affect the analysis results. However, Zhang *et al*. used missing data on patients with depression (28 people) and complete data on patients with sarcopenia (109 people) to calculate the prevalence, so their prevalence was incorrect.

**Table 1:** Two sets of data from the original study

	Depression	Sarcopenia	Prevalence
Data from table	28	94	29.8%
Data from text	32	109	29.8%

Second, Although Nyaga *et al*. proposed that a meta‐analysis of single‐group interest rates using the *metaprop* command may be more realistic, *metan* command is also widely used.^3^, ^4^ To verify whether the two commands would lead to different results in our study, we replotted the forest plot using *metapro* command (*Figure* 1). The result showed that the overall prevalence of depression in patients with sarcopenia was 0.283 (95% CI: 0.210–0.355), significant heterogeneity was noted (*P* < 0.001; *I* ^2^ = 92.112%). This result is consistent with the outcome of using the *metan* command (prevalence was 0.28 (95% CI: 0.21–0.36; *I* ^2^ = 92.2%), which means that both commands can be used in our study. The data extracted by Zhang *et al*. were incorrect, they expanded the sample size of sarcopenia, underestimated the prevalence of depression in two studies (Olgun Yazar's and Hsu's) (*Figure* S4), and the calculated prevalence (0.256) was lower than ours. Therefore, the differences were caused by their erroneous data extraction rather than the different command of the Stata software.

**Figure 1:**
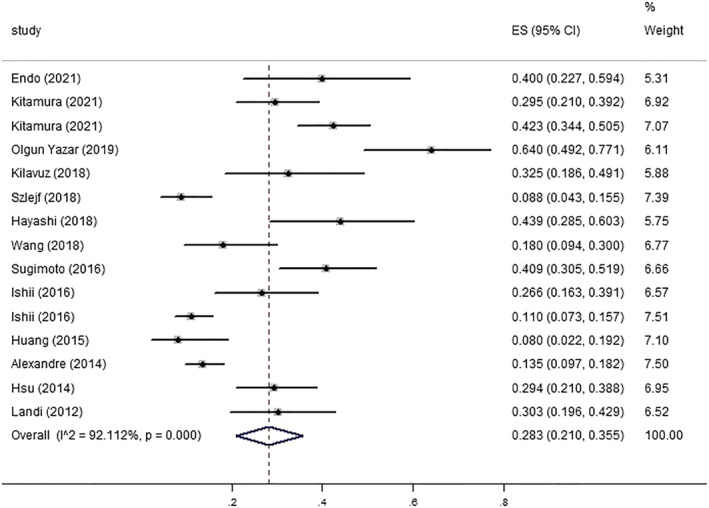
Forest plot of prevalence of depression in sarcopenia using *metapro* command.

Third, it is true that the results of the analysis incorporating data may be more relevant and accurate. However, we found that the results for the overall data were consistent with the results for males and females separately, and we thought there were other advantages to show them separately. The differences between genders can be displayed, ‘The findings showed that the prevalence and OR of depression in women with sarcopenia were higher than those in men with sarcopenia, perhaps because women are more likely to suffer from sarcopenia and depression than men’,^2^ which providing a basis for possible further research.

In addition, we exactly considered doing meta‐regression at the beginning. However, after consulting with a methodologist at the Chinese Center for Evidence‐Based Medicine, we found the meta‐regression was not feasible. In the process of meta‐regression, the number of variables cannot exceed one‐fifth of the number of included studies. If the variable is nominal, it must be split into a dummy variable for analysis, and the number of dummy variables cannot exceed the included studies. If we conduct meta‐regression, there are six nominal variables in our analysis, and the number of dummy variables is more than 15, so we cannot conduct meta‐regression. Therefore, we did a subgroup analysis to investigate whether covariates affected the pooled effect.

At last, thanks for pointing out the lack of assessment of publication bias. We have performed a funnel plot, and publication bias was detected among the studies included for prevalence analysis (*Figure* 2). In contrast, no publication bias was seen among the studies included for ORs analysis (*Figure* 3).

**Figure 2:**
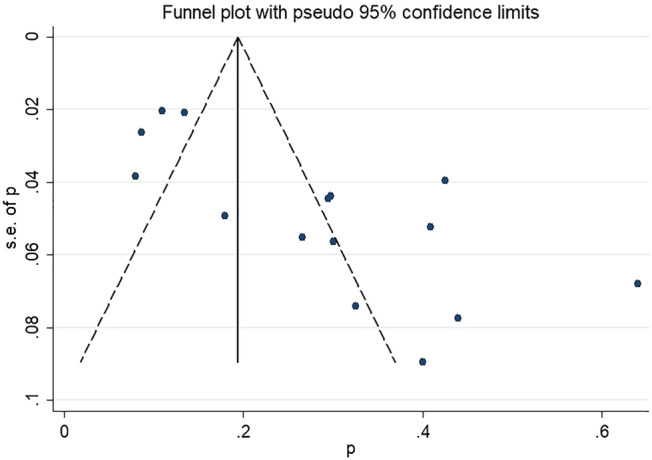
Funnel plot of prevalence of depression in sarcopenia.

**Figure 3:**
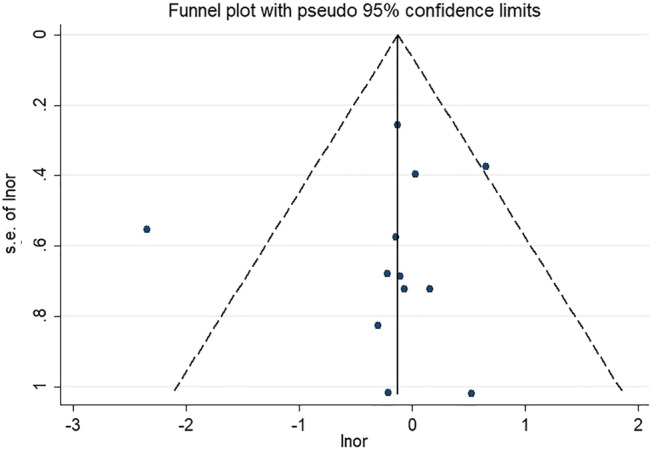
Funnel plot of the adjusted odds ratios (ORs) between sarcopenia and depression.

## Supporting information


**Figure S1.** The Table 2 of Olgun Yazar’ study.

**Figure S2.** Text description of the results of Hsu’ study.

**Figure S3.** The Table 1 of Hsu’ study.

**Figure S4.** Zhang's incorrect forest plot.
