# Supplementary figures and images for: The authors reply: Letter on: "Prevalence of depression in patients with sarcopenia and correlation between the two diseases: systematic review and meta‐analysis" by Li et al
Source: J Cachexia Sarcopenia Muscle. 2022 May 1;13(4):2254–6. doi: 10.1002/jcsm.13008 (PMC9397551; doi:10.1002/jcsm.13008)

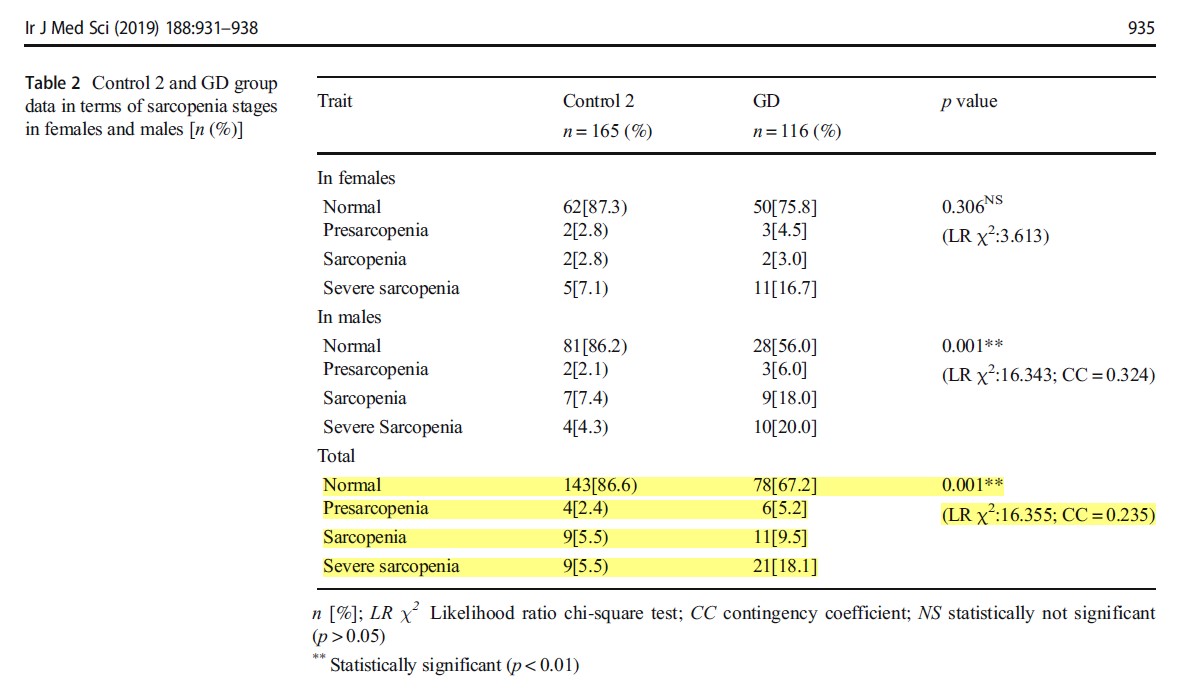

Supplement: Supplementary file 1 — Figure S1. The Table 2 of Olgun Yazar’ study. [file JCSM-13-2254-s001.jpg]

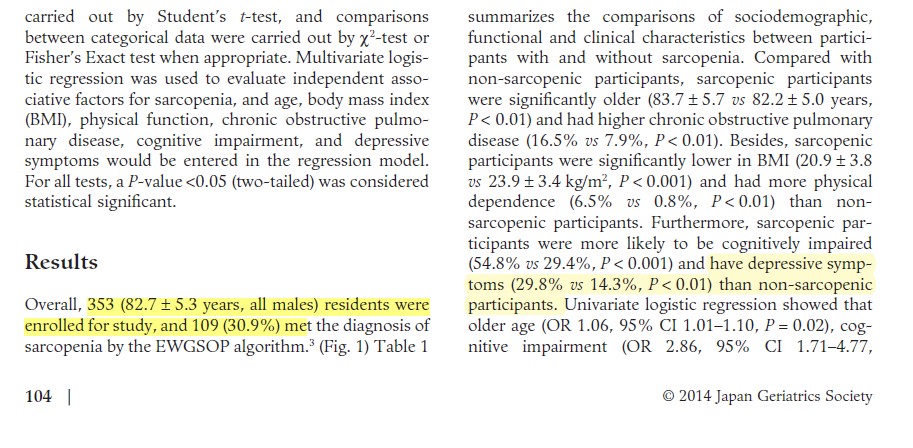

Supplement: Supplementary file 2 — Figure S2. Text description of the results of Hsu’ study. [file JCSM-13-2254-s004.jpg]

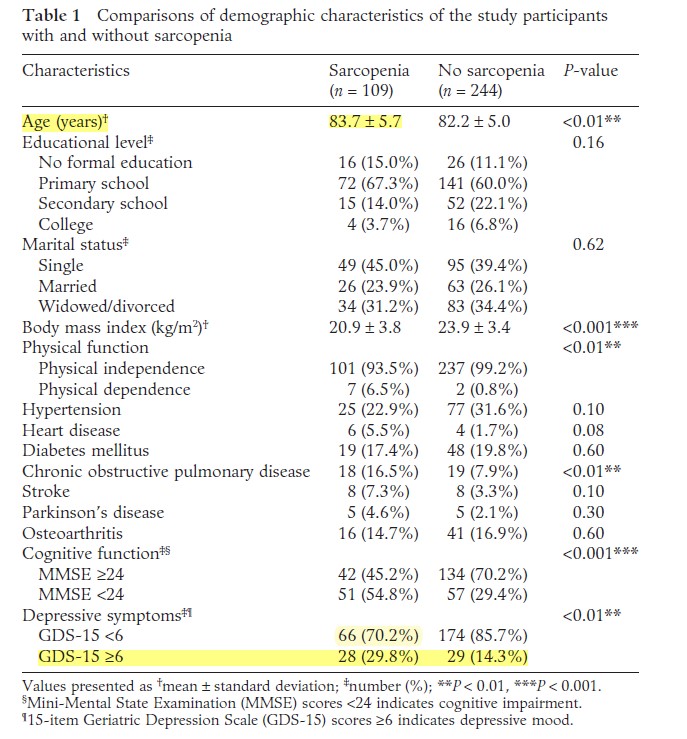

Supplement: Supplementary file 3 — Figure S3. The Table 1 of Hsu’ study. [file JCSM-13-2254-s002.jpg]

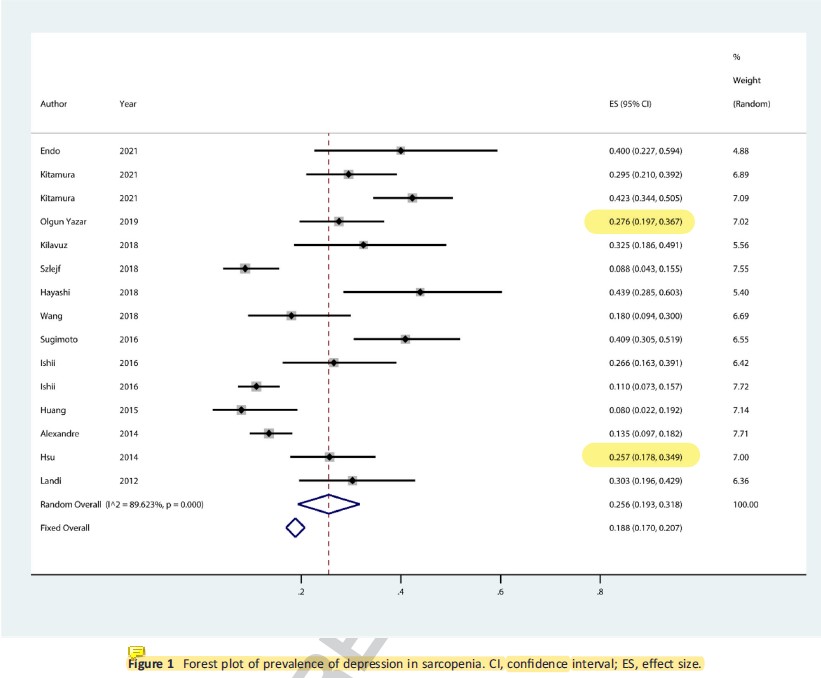

Supplement: Supplementary file 4 — Figure S4. Zhang's incorrect forest plot. [file JCSM-13-2254-s003.jpg]
